# The advanced lung cancer inflammation index predicts long-term outcomes in patients with hypertension: National health and nutrition examination study, 1999–2014

**DOI:** 10.3389/fnut.2022.989914

**Published:** 2022-10-25

**Authors:** Yanbin Zhang, Yuxiong Pan, Jiabin Tu, Lihua Liao, Shuqiong Lin, Kaihong Chen, Shan Ding, Guitao Xiao

**Affiliations:** ^1^Longyan First Affiliated Hospital of Fujian Medical University, Longyan, China; ^2^The Third Clinical Medical College, Fujian Medical University, Fuzhou, China; ^3^Zhangzhou Affiliated Hospital of Fujian Medical University, Zhangzhou, China; ^4^The People’s Hospital of Longyan, Longyan, China

**Keywords:** hypertension, nutrition, inflammation, advanced lung cancer inflammation index, NHANES

## Abstract

**Background:**

Malnutrition and systemic inflammation are associated with poor outcomes in patients with hypertension, and the two often coexist. However, few studies have combined nutritional and inflammatory status to assess the prognosis of patients with hypertension. The present study aimed to investigate the association between advanced lung cancer inflammation index (ALI), as a factor assessment the nutritional and inflammatory status, and long-term all-cause mortality of patients with hypertension.

**Materials and methods:**

Data from the National Health and Nutrition Examination Survey (NHANES) 1999–2014 with mortality follow-up through December 31, 2015, were analyzed. A total of 15,681 participants were evaluated. The patients were grouped based on the ALI tertiles as follows: T1 (ALI ≤ 49.41, *n* = 5,222), T2 (ALI > 49.41 and ≤ 76.29, *n* = 5,221), and T3 (ALI > 76.29, *n* = 5,237) groups. Survival curves and Cox regression analysis based on the NHANES recommended weights were used to assess the relationship between nutritional and inflammatory status and long-term all-cause mortality.

**Results:**

Advanced lung cancer inflammation index was significantly associated with long-term all-cause mortality in patients with hypertension. After adjustment for related factors, the T2 [hazard ratio (HR): 0.69, 95% confidence interval (CI): 0.58–0.83; P < 0.001) and T3 (HR: 0.59, 95% CI: 0.47–0.74; P < 0.001) groups were significantly associated with a decreased risk of all-cause mortality compared to the lower ALI level group (T1).

**Conclusion:**

Advanced lung cancer inflammation index was a comprehensive index of nutrition and inflammation and an independent significant prognostic factor in hypertension patients in the American community. Systemic inflammatory and nutritional status assessment and monitoring are essential for the health of hypertensive patients.

## Introduction

Hypertension is the most common cardiovascular disease affecting about 1 billion people worldwide (1, 2). It is the leading cause of global morbidity and mortality. About 51% of stroke deaths and 45% of cardiovascular deaths are caused by hypertension (3, 4). Despite the widespread availability of effective treatments, hypertension control in the American community remains suboptimal (5).

Studies have shown that malnutrition affects the prognosis of people with hypertension (6). Similarly, inflammatory markers, such as high sensitivity C-reactive protein and interleukin-6, can also effectively predict the development of hypertension (7). However, malnutrition and inflammation often interact with each other. Inflammation stimulates muscle protein hydrolysis and catabolism, inhibits repair mechanisms, and leads to muscle mass loss, while malnutrition can damage the immune system of the elderly, make them vulnerable to infection, and trigger the state of inflammation (8, 9). Therefore, utilizing the nutritional or inflammatory markers alone to evaluate the prognosis of patients with hypertension is not accurate.

Advanced lung cancer inflammation index (ALI) is calculated using the following formula: BMI (kg/m^2^) × serum albumin level (g/dL)/(neutrophil count/lymphocyte count), which can reflect both the nutritional and inflammatory patient status. It was originally used to assess inflammation and nutritional status in lung cancer patients and has been associated with all-cause mortality in lung cancer patients (10). ALI has since been promoted for many diseases that are also affected by both nutrition and inflammation, such as Crohn’s disease (11), coronary artery disease (12) and heart failure (13). Hypertension is also affected by nutrition and inflammation (14, 15). However, rare studies have investigated the relationship between ALI and the prognosis of patients with hypertension.

The purpose of the present study was to investigate the relationship between ALI and long-term all-cause mortality of patients with hypertension and to provide a reference for the diagnosis, treatment, and management of patients with hypertension in American community.

## Materials and methods

### Study population

The study data were extracted from the National Health and Nutrition Examination Survey (NHANES), which collects data every 2 years and is part of the United States Public Health Surveillance program. The Centers for Disease Control and Prevention initiated NHANES and use a complex, multistage, probability sampling design to produce a nationally representative sample of non-hospitalized population in the United States. NHANES was approved by the NCHS Research Ethics Review Board.^1^

**FIGURE 1:**
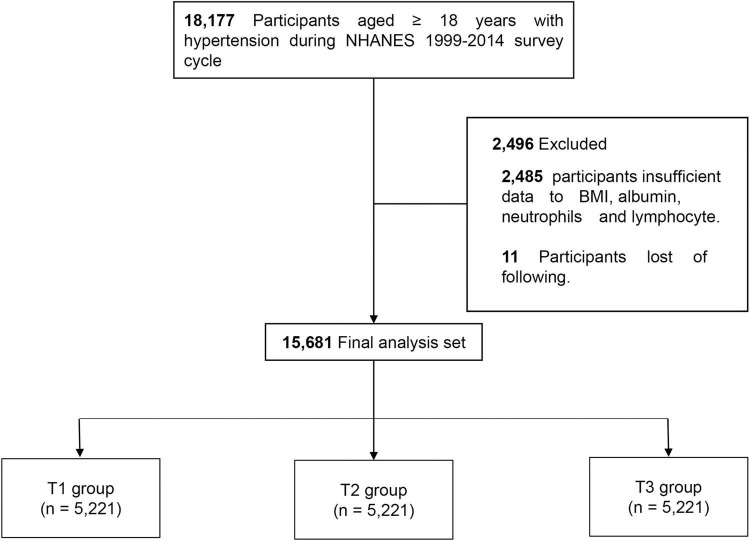
Flowchart of study design.

National Health and Nutrition Examination Survey 1999–2014 participants with hypertension who were ≥18 years of age were included in the study (*n* = 18,177). Of these participants, 2,485 were excluded due to missing body mass index (BMI), albumin, neutrophil, and lymphocyte data, and 11 were excluded due to loss to follow-up. A total of 15,681 patients were enrolled in the study and were divided into three groups based on ALI tertiles as follows: T1 (≤49.41), T2 (>49.41 and ≤76.29), and T3 (>76.29) (Figure 1).

### Hypertension assessment

Hypertension diagnosis was based on an affirmative response to the question: “Has a doctor or other health professional ever told you that you had hypertension?” In addition, subjects with systolic blood pressure (SBP) of ≥140 mmHg and/or diastolic blood pressure (DBP) of ≥90 mmHg were also considered hypertensive. Participants who were currently taking blood pressure medications were also considered hypertensive. In addition, the patients were divided into grade 1 hypertension (140 ≤ SBP < 160 mmHg, 90 < DBP < 100), grade 2 hypertension (160 ≤ SBP < 180 mmHg, 100 ≤ DBP ≤ 110) and grade 3 hypertension (SBP > 180 mmHg, DBP > 110) according to their blood pressure values (16).

### Variables of interest definitions

Age, sex, race, smoking status, alcohol use, BMI, antihypertensive drug use, and history of stroke, congestive heart failure (CHF), coronary heart disease (CHD), chronic kidney diseases (CKD), diabetes mellitus (DM), and liver disease were self-reported. CKD was also defined as an Estimated glomerular filtration rate (eGFR) < 90 ml/min per 1.73 m^2^. eGFR was used to estimate by the CKD Epidemiological Collaboration (17). Diagnosis of comorbidities was based on an affirmative response to the following question: “Has a doctor or other health professional ever told you that you had CHF, CHD, CKD, DM, or liver disease?”

### Primary outcome

Mortality status was determined based on a probabilistic record match with the National Death Index using demographic identifiers. The primary outcome was all-cause mortality. Mortality follow-up data for participants from NHANES 1999–2014 were available through December 31, 2015.

### Statistical analyses

The weighting calculation for specific groups was carried out according to the weights provided on the official NHANES website. The continuous variables were expressed as the means ± standard deviation. Categorical variables were expressed as counts (percentages). Baseline characteristics among the three groups were compared using ANOVA for continuous variables and χ^2^ test for categorical variables. To eliminate the influences of extreme values, all data were processed and winsorized at the 1 and 99% quantiles.

Kaplan-Meier estimates based on the NHANES-recommended weights were used to analyze the relationship between ALI and all-cause mortality. Univariate and multivariate Cox regression analyses based on the NHANES-recommended weights were conducted to investigate the association between ALI and all-cause mortality. Model 1 was a crude unadjusted model of potential confounders. Model 2 was adjusted for age, sex, and race/ethnicity. Model 3 was further adjusted for age, gender, race/ethnicity, education, smoke status, alcohol use, BMI, CHF, CHD, DM, CKD, stroke, liver disease, and antihypertensive drug use. The relationship between ALI and mortality was further explored in different subgroups, including age, sex, obesity, and hypertension grade. A possible non-linear relationship between ALI and mortality was assessed using a restricted cubic spline (RCS) Cox model. RCS was also used to visualize the relationship between ALI and mortality in participants with different hypertension grades.

All analyses were performed using R software (version 4.0.3; R Foundation for Statistical Computing, Vienna, Austria). A two-sided *P*-value of <0.05 indicated statistical significance for all analyses.

## Results

### Patient characteristics

A total of 15,681 participants with hypertension were enrolled in the study, including 7,711 males and 7,970 females (mean age: 56.6 ± 0.2 years). Participants were younger (T1 group: 61.3 ± 0.3 vs. T2 group: 55.8 ± 0.4 vs. T3 group: 53.3 ± 0.3 years) and had a higher BMI (T1 group: 27.4 ± 0.1 vs. T2 group: 30.7 ± 0.1 vs. T3 group: 33.6 ± 0.1) in the group with a higher ALI. As ALI increased, the proportion of smokers (T1 group: 54.7% vs. T2 group: 50.6% vs. T3 group: 47.5%) and drinkers (T1 group: 71.3% vs. T2 group: 68.0% vs. T3 group: 67.3%) decreased gradually and was less likely to be combined with stroke (T1 group: 7.3% vs. T2 group: 5.0% vs. T3 group: 4.4%), CHD (T1 group: 9.5% vs. T2 group: 6.6% vs. T3 group: 4.9%) and CHF (T1 group: 6.6% vs. T2 group: 4.4% vs. T3 group: 3.7%), while CKD (T1 group: 14.7% vs. T2 group:15.8% vs. T3 group: 16.6%) and antihypertensive drug use (T1 group: 84.4% vs. T2 group: 80.1% vs. T3 group: 80.3%) was lower. The baseline characteristics of the participants are shown in Table 1.

**TABLE 1:** Baseline study population characteristics (weighted).

	Overall (*n* = 15,681)	T1 group (*n* = 5,227)	T2 group (*n* = 5,227)	T3 group (*n* = 5,227)	*P*-value
Age, mean (SE), years	56.8 ± 0.2	61.3 ± 0.3	55.8 ± 0.4	53.3 ± 0.3	<0.001
Female [*n* (%)]	7,970 (51.3)	2,473 (50.4)	2,642 (49.9)	2,855 (53.9)	0.002
**Race [*n* (%)]**					
Mexican American	2,340 (5.4)	678 (4.2)	929 (6.4)	733 (5.5)	<0.001
Other hispanic	1,019 (4.1)	303 (3.2)	364 (4.7)	352 (4.4)	
Non-hispanic white	7,649 (72.6)	3,157 (80.5)	2,600 (73.9)	1,892 (63.0)	
Non-hispanic black	3,807 (12.9)	800 (7.5)	1,037 (10.0)	1,970 (21.6)	
Other race	866 (5.0)	289 (4.6)	297 (5.0)	280 (5.4)	
Smoking status [*n* (%)]	7,819 (51.0)	2,824 (54.7)	2,586 (50.6)	2,409 (47.5)	<0.001
Alcohol* [*n* (%)]	5,796 (68.9)	1,966 (71.3)	1,954 (68.0)	1,876 (67.3)	0.028
BMI (SE)	30.6 ± 0.1	27.4 ± 0.1	30.7 ± 0.1	33.6 ± 0.1	<0.001
Stroke [*n* (%)]	1,059 (5.6)	461 (7.3)	331 (5.0)	267 (4.4)	<0.001
DM [*n* (%)]	3,080 (15.7)	1,003 (14.7)	1,014 (15.8)	1,063 (16.6)	0.092
CHD [*n* (%)]	1,194 (7.0)	531 (9.5)	397 (6.6)	266 (4.9)	<0.001
CHF [*n* (%)]	933 (4.9)	422 (6.6)	283 (4.4)	228 (3.7)	<0.001
CKD [*n* (%)]	4,867 (74.4)	2,121 (66.4)	1,494 (76.6)	1,252 (80.1)	<0.001
Liver disease [*n* (%)]	702 (4.5)	234 (4.2)	213 (4.2)	255 (5.0)	0.572
Hypotensive drugs [*n* (%)]	10,806 (81.6)	3,645 (84.4)	3,546 (80.1)	3,615 (80.3)	<0.001

T1 group (ALI ≤ 49.41), T2 group (ALI > 49.41 and ≤ 76.29), T3 group (ALI > 76.29).

*The average number of drinks consumed per day over the past 12 months.

BMI, body mass index; DM, diabetes mellitus; CHD, coronary heart disease; CHF, congestive heart failure; CKD, Chronic kidney disease.

### Advanced lung cancer inflammation index and all-cause mortality

**FIGURE 2:**
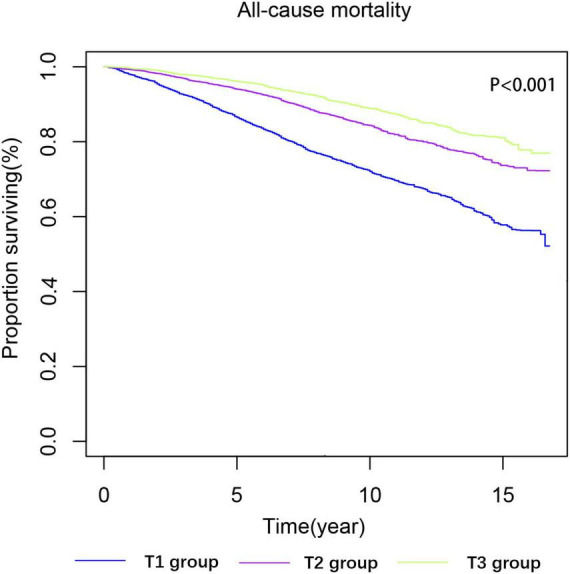
Kaplan-Meier survival estimates for long-term all-cause mortality (weighted).

**FIGURE 3:**
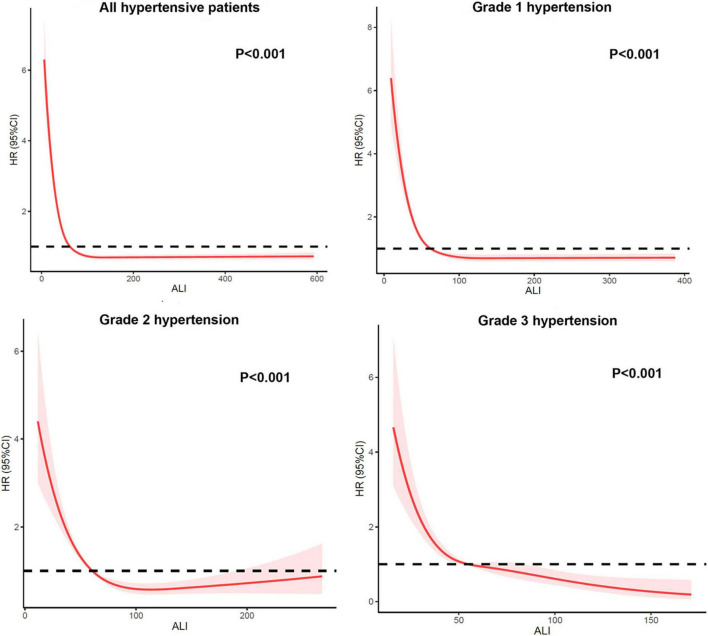
Restricted cubic spline of hazard ratios (HRs) and 95% CI for the association between ALI score (continuous) and all-cause mortality (unweighted).

Kaplan-Meier survival analysis curves showed that the long-term mortality was lower in the groups with a higher ALI (*P*-log rank < 0.001, Figure 2). Univariate Cox proportional risk analysis showed that the risk of all-cause mortality decreased by 1% for each unit of increase in ALI, while the risk of all-cause mortality decreased by 12% for every 10 units of increase in ALI. Compared to the T1 group, the T2 [hazard ratio (HR): 0.53, 95% confidence interval (CI): 0.48–0.58; *P* < 0.001) and T3 (HR: 0.36, 95% CI: 0.31–0.41; *P* < 0.001] groups had a lower risk of all-cause mortality. After a full adjustment for potential confounders, the risk of all-cause mortality decreased by 1% for every unit of increase in ALI and 6% for every 10 units of increase. The T2 (HR: 0.69, 95% CI: 0.58–0.83; *P* < 0.001) and T3 (HR: 0.59, 95% CI: 0.47–0.74; *P* < 0.001) groups had a lower risk of death than the T1 group (Table 2). The RCS model showed a non-linear relationship between ALI and all-cause mortality (*P* < 0.001, Figure 3).

**TABLE 2:** Associations between advanced lung cancer inflammation index (ALI) and all-cause mortality in NHANES 1999–2014 followed through 2015.

Variable	Model 1			Model 2			Model 3		
	HR	95% CI	*P*-value	HR	95% CI	*P*-value	HR	95% CI	*P*-value
**Continuous variables**									
ALI per 1 U	0.99	0.98–0.99	*P* < 0.001	0.99	0.99–1.00	0.001	0.99	0.99–1.00	0.002
ALI per 10 U	0.88	0.85–0.91	*P* < 0.001	0.95	0.92–0.98	0.003	0.94	0.90–0.98	0.002
**Tripartite variable**									
Group 1	Ref	–	–	Ref	–	–	Ref	–	–
Group 2	0.53	0.48–0.58	<0.001	0.71	0.65–0.78	<0.001	0.69	0.58–0.83	<0.001
Group 3	0.36	0.31–0.41	<0.001	0.59	0.51–0.67	<0.001	0.59	0.47– 0.74	<0.001

Model 1: No adjusted.

Model 2: Adjusted by age, gender, and race/ethnicity.

Model 3: Adjusted by age, gender, race/ethnicity, education, smoke status, alcohol, BMI, CHF, CHD, DM, CKD, stroke, Liver disease, and Antihypertensive drugs.

### Advanced lung cancer inflammation index and all-cause mortality in different grade of hypertension

When participants were stratified by hypertension grade, the risk of all-cause mortality in patients with grade 2 hypertension was also lower in the group with a higher ALI (T2 group: HR: 0.72, 95% CI: 0.53–0.98; *P* = 0.039; T3 group: HR: 0.47, 95% CI: 0.29–0.77; *P* = 0.003). The T3 group (HR: 0.40, 95% CI: 0.22–0.74; *P* = 0.003) remained with a lower risk of all-cause mortality than the T1 group in patients with grade 2 hypertension. The T3 group (HR: 0.13, 95% CI: 0.03–0.61; *P* = 0.004) had a lower risk of mortality than the T1 group in patients with grade 3 hypertension (Table 3). The RCS model also showed consistent ALI results for all-cause mortality when stratified by hypertension grade (Figure 3).

**TABLE 3:** All-cause mortality hazard ratios (HRs) in different grade of hypertension.

	All-cause mortality		
Variable	HR	95% CI	*P*-value
**Grade 1 hypertension**			
T1 group	Ref	–	–
T2 group	0.72	0.53–0.98	0.039
T3 group	0.47	0.29–0.77	0.003
**Grade 2 hypertension**			
T1 group	Ref	–	–
T2 group	0.61	0.36–1.02	0.060
T3 group	0.40	0.22–0.74	0.003
**Grade 3 hypertension**			
T1 group	Ref	–	–
T2 group	0.51	0.18–1.49	0.221
T3 group	0.13	0.03–0.61	0.004

Adjusted by age, gender, race/ethnicity, smoke status, alcohol, BMI, CHF, CHD, DM, CKD, stroke, Liver disease, and Hypotensive drugs.

### Subgroups

**FIGURE 4:**
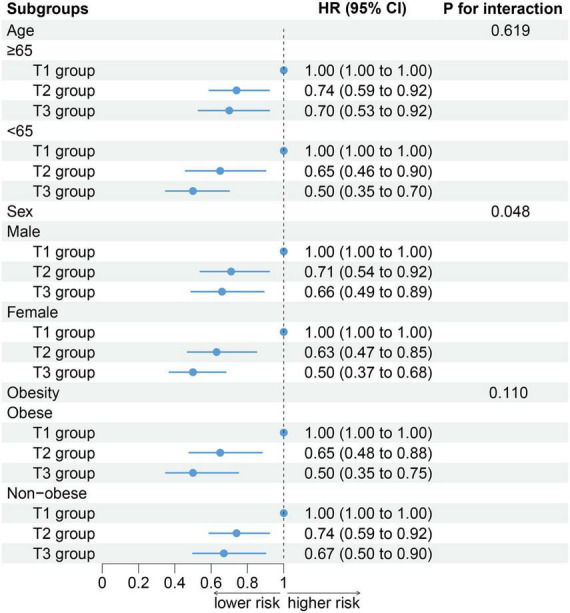
The association between advanced lung cancer inflammation index (ALI) and all-cause mortality by selected subgroups.

Subgroup analysis showed a significant interaction between sex and ALI (*P* for interaction = 0.048). All-cause mortality decreased in the male subgroup with the increase in ALI (T2 group: HR: 0.70, 95% CI: 0.54–0.92; *P* = 0.009; T3 group: HR: 0.66, 95% CI: 0.49–0.89; *P* = 0.006). These results were even more significant in the female subgroup (T2 group: HR: 0.63, 95% CI: 0.47–0.85; *P* = 0.003; T3 group: HR: 0.50, 95% CI: 0.37–0.68; *P* < 0.001). However, similar interactions were not observed in the age (*P* for interaction = 0.619) and obesity (*P* for interaction = 0.110) subgroups (Figure 4).

## Discussion

A comprehensive index ALI was used to assess the combined nutritional and inflammatory status of American community population and to further explore the prognosis of patients with hypertension in a retrospective study. The results suggested that the group with a higher ALI had a lower risk of all-cause mortality in patients with hypertension even after adjustment for potential confounding factors. Moreover, stratified analysis of hypertension classification demonstrated that the risk of death remained decreased in the higher ALI groups.

Hypertension is one of the most prevalent chronic diseases affecting over one-third of adults worldwide in 2010 (18). It has become the main contributor to mortality and disability (19). An estimated 7.7–10.4 million annual deaths are attributable to elevated blood pressure levels (20). The global burden of hypertension is rising despite the availability of anti-blood pressure drugs and interventions such as lifestyle modification. Epidemiological studies suggest that the global disease burden attributable to hypertension was 7% in 2010 and is predicted to rise to as high as 29.2% by 2025 (21). The impact of nutritional status and systemic inflammation on the development and prognosis of hypertension was noted. Yang et al. have reported that malnutrition was closely associated with both long-term cardiovascular mortality and all-cause mortality among hypertensive patients (6). Epidemiological studies have indicated that the presence of a chronic low-grade inflammatory status also anticipates the future development of hypertension (22). Even though BMI, albumin level, as well as neutrophil to lymphocyte ratio, which are involved in malnutrition and inflammation, are considered to be independent risk factors or predictors in multiple diseases (23–25), growing evidence has demonstrated a complex interplay between malnutrition and inflammation. It has been reported that inflammation affects malnutrition and adverse outcomes through different pathways (26, 27). Observations by Mujico et al. have suggested that malnutrition played a key role in the development of associated systemic inflammation and related pathologies (28). In addition, more and more studies have integrated nutrition and inflammation to assess disease development and prognosis (29–31). Importantly, ALI, a comprehensive evaluation index based on nutrition and inflammation, was associated with poor prognosis in several types of cancers, as well as in heart failure (10, 13, 32, 33). Hypertension is well-known to trigger incident heart failure. Therefore, ALI was used to predict the hypertension prognosis and confirmed that the long-term mortality decreased significantly in groups with a higher ALI.

Our results showed that the risk of long-term all-cause mortality in hypertensive patients decreased as ALI increased. In previous studies on ALI, higher ALI was associated with a lower risk of all-cause death in patients with diseases related to nutrition and inflammation. The result of Jafri et al. shown that patients with non-small cell lung cancer with higher ALI had a decreased risk of all-cause death. A study of Crohn’s disease showed that lower ALI levels were an independent risk factor for reoperation after bowel resection (11). In addition, studies have shown a decreased risk of all-cause death and readmission in HF patients with higher ALI levels (13). Although the populations studied varied, non-small cell lung cancer, Crohn’s disease, and heart failure can all lead to changes in nutrition and inflammation levels in patients as can hypertension. Therefore, it is feasible to use ALI to assess nutritional and inflammatory levels in hypertensive patients and explore their association with long-term all-cause mortality in hypertensive patients, and we have fully adjusted for potential influencing factors, our results are reliable.

Stratified analysis of hypertension grades showed that the risk of all-cause mortality predicted by the T3 group ALI decreased more in grade 3 hypertension. That is, the risk of death is more closely associated with inflammation and malnutrition in high grade hypertension. Studies have suggested that hypoalbuminemia in hypertensive patients likely occurs due to endothelial dysfunction, capillary leakage, as well as albumin loss in renal tubules, which are directly related to blood pressure levels (34–37). In addition, serum albumin levels are influenced by the nutritional status, inflammatory state, and other factors, further affecting patient outcomes (34, 38). In addition, Yasanari et al. have mentioned that the higher the blood pressure, the greater the degree of oxidative stress in leukocytes (39). It has been reported that oxidative stress and inflammation affected both micro- and macrovascular functions, promoting a vicious cycle between increased blood pressure, vascular remodeling, and stiffness (40). Previous studies have suggested that the mortality rises with a higher classification of hypertensive severity (41, 42), almost doubling the risk of death with systolic blood pressure rising by as much as 20 mmHg and diastolic blood pressure by 10 mmHg (43). Therefore, it is very important to monitor the ALI index to predict the hypertension prognosis, especially in grade 3 hypertension.

Further subgroup analysis of age, sex, and obesity showed that the ability of ALI to predict all-cause mortality was similar and stable in different age and obesity subgroups. However, the risk of mortality predicted by ALI in hypertensive patients was significantly different between females and males. The protective effect of ALI was higher in the female subgroup than the male subgroup. Although the mechanism is not clear, we think it may have to do with biological differences between men and women. Malnutrition and inflammation are independent risk factors for early menopause in women (44, 45). There is a lot of evidence that early menopause increases a woman’s risk of all-cause death, cardiovascular death (46). Therefore, women may be more affected by malnutrition and inflammation than men, which may explain our results.

As a comprehensive assessment of patient nutrition and inflammation levels, ALI is associated with long-term all-cause mortality in hypertensive patients. Studies have shown that the benefit of nutritional support is affected by inflammation (47). Therefore, a measure that can improve both nutritional status and inflammation levels is needed. We noticed that many nutritional supplements have anti-inflammatory effects, such as resveratrol and anthocyanins (48, 49). These supplements can not only improve the nutritional status of patients, but also reduce the level of inflammation in patients. Although the results of a randomized controlled study suggest that a Mediterranean diet may prevent an increase in ALI in lung cancer patients (50). Many studies have suggested that the Mediterranean diet is beneficial for people with high blood pressure (51, 52). We recommend that patients with hypertension in the community try the Mediterranean diet, and consider the use of nutritional supplements, which may be helpful for the long-term prognosis of patients with hypertension.

### Limitations

The present study had some limitations. Some data for hypertension diagnosis and comorbidities came from self-reported questionnaires, which may be different from the actual patient status. Second, we were unable to obtain information on changes in patients’ physical status after baseline data collection, and the nutritional and inflammatory patient status cannot be evaluated based on the data from only one blood draw. In addition, the retrospective nature of the study only allowed to conclude that the ALI index was associated with all-cause death in hypertensive patients without demonstrating causality.

## Conclusion

The present study found that ALI, a comprehensive index of nutrition and inflammation, was an independent significant prognostic factor in hypertension patients in the American community. Systemic inflammatory and nutritional status assessment and monitoring are essential for hypertensive patient health. Future studies to develop and validate better risk assessment tools are needed to assess the combined nutritional and inflammatory status in patients with hypertension.

## Data availability statement

The raw data supporting the conclusions of this article will be made available by the authors, without undue reservation.

## Author contributions

YZ was guarantor of this work and, as such, had full access to all the data in the study and takes responsibility for the integrity of the data and the accuracy of the data analysis. YP conducted analyses and wrote the first draft of the manuscript. KC, SD, and GX designed the research. JT, LL, SL, KC, SD, and GX revised the manuscript. All authors contributed to the article and approved the submitted version.

## Footnotes

1
https://www.cdc.gov/nchs/nhanes/irba98.htm
